# Biological Profiling Enables Rapid Mechanistic Classification of Phenotypic Screening Hits and Identification of KatG Activation-Dependent Pyridine Carboxamide Prodrugs With Activity Against *Mycobacterium tuberculosis*

**DOI:** 10.3389/fcimb.2020.582416

**Published:** 2020-11-13

**Authors:** Melissa D. Chengalroyen, Audrey Jordaan, Ronnett Seldon, Thomas Ioerger, Scott G. Franzblau, Mohamed Nasr, Digby F. Warner, Valerie Mizrahi

**Affiliations:** ^1^ SAMRC/NHLS/UCT Molecular Mycobacteriology Research Unit & DST/NRF Centre of Excellence for Biomedical TB Research, Institute of Infectious Disease and Molecular Medicine & Department of Pathology, University of Cape Town, Cape Town, South Africa; ^2^ H3D Drug Discovery and Development Centre, Department of Chemistry, University of Cape Town, Cape Town, South Africa; ^3^ Department of Computer Science and Engineering, Texas A&M University, College Station, TX, United States; ^4^ Institute for Tuberculosis Research, Department of Medicinal Chemistry and Pharmacognosy, College of Pharmacy, University of Illinois at Chicago, Chicago, IL, United States; ^5^ Division of AIDS, NIAID, National Institutes of Health, Bethesda, MD, United States; ^6^ Wellcome Centre for Infectious Diseases Research in Africa, University of Cape Town, Cape Town, South Africa

**Keywords:** antimycobacterial, catalase, drug resistance, isoniazid, KaG, luciferase, tuberculosis

## Abstract

Compounds with novel modes of action are urgently needed to develop effective combination therapies for the treatment of tuberculosis. In this study, a series of compounds was evaluated for activity against replicating *Mycobacterium tuberculosis* and Vero cell line toxicity. Fourteen of the compounds with *in vitro* activities in the low micrometer range and a favorable selectivity index were classified using reporter strains of *M. tuberculosis* which showed that six interfered with cell wall metabolism and one disrupted DNA metabolism. Counter-screening against strains carrying mutations in promiscuous drug targets argued against DprE1 and MmpL3 as hits of any of the cell wall actives and eliminated the cytochrome *bc*_1_ complex as a target of any of the compounds. Instead, whole-genome sequencing of spontaneous resistant mutants and/or counter-screening against common isoniazid-resistant mutants of *M. tuberculosis* revealed that four of the six cell wall-active compounds, all pyridine carboxamide analogues, were metabolized by KatG to form InhA inhibitors. Resistance to two of these compounds was associated with mutations in *katG* that did not confer cross-resistance to isoniazid. Of the remaining seven compounds, low-level resistance to one was associated with an inactivating mutation in Rv0678, the regulator of the MmpS5-MmpL5 system, which has been implicated in non-specific efflux of multiple chemotypes. Another mapped to the mycothiol-dependent reductase, Rv2466c, suggesting a prodrug mechanism of action in that case. The inability to isolate spontaneous resistant mutants to the seven remaining compounds suggests that they act *via* mechanisms which have yet to be elucidated.

## Introduction

Globally, it is estimated that the incidence of tuberculosis (TB) is declining by ~2% each year (WHO, 2019). However, the ongoing evolution and spread of strains of *Mycobacterium tuberculosis* (Mtb) resistant to first- and second-line anti-TB drugs and consequent increase in the incidence of drug-resistant TB threatens to diminish this decline and underscores the need for new drugs with novel mechanisms of action. In response to this need, a TB drug pipeline that is reasonably well-populated with new and repurposed anti-tubercular agents has been established (https://www.newtbdrugs.org/). Encouragingly, the discovery stage of the pipeline includes molecules that act on a more diverse range of essential cellular processes in Mtb which extends beyond the classical pathways of cell wall biosynthesis, transcription, energy metabolism, DNA replication, and protein synthesis targeted by existing first- and second-line TB drugs (Evans and Mizrahi, 2018; Wellington and Hung, 2018; Lienhardt and Raviglione, 2020). The challenges posed for compound penetration across the mycobacterial cell envelope, coupled with the ability of Mtb to metabolize and efflux small molecules have confounded target-led approaches to drug discovery. As a result, phenotypic approaches have predominated, with whole-cell assays having been developed and applied in high-throughput screens to identify molecules with inhibitory activity against Mtb under conditions which support bacillary replication or non-replicating persistence and are modeled on those encountered during human infection (Deb et al., 2009; Ioerger et al., 2013; Gold et al., 2015; Gold and Nathan, 2017; Huang et al., 2018; Parish, 2020). However, this approach is not without its own challenges, the most significant of which is mechanism-of-action (MoA) elucidation of compounds with whole-cell activity against Mtb. This process can be difficult and time-consuming (Singh and Mizrahi, 2017) and yet, is critically important for ensuring that the scarce resources available for hit expansion and/or hit-to-lead progression are deployed most judiciously. As a result, considerable effort has been placed on developing and applying biological assays that enable broad mechanistic classification—and hence, triage—of phenotypic screening hits that pass a certain threshold of cytotoxicity. Assays of this type are particularly useful for rapidly identifying chemotypes that act on promiscuous drug targets such as DprE1, MmpL3, Pks13, or QcrB for which multiple inhibitory scaffolds already exist (Cole, 2016; Lee and Pethe, 2018).

In this paper, we describe the application of this approach to the characterization of a set of 14 compounds selected on the basis of an anti-TB pharmacophore centered virtual screen conducted by the Division of AIDS at the NIAID.

## Materials and Methods

### Bacterial Strains, Culture Conditions, and Media

The strains employed in this study are described in **Supplementary Table 1**. Mycobacterial strains were cultured in various media depending on the assay conducted. 7H9 OADC was prepared by supplementing Middlebrook 7H9 (Difco) with 100 ml oleic acid-albumin-dextrose-catalase (OADC) enrichment (Difco), 2g/L glycerol, and 2.5 ml 25% Tween 80. 7H9 ADC medium was prepared by supplementing Middlebrook 7H9 with 100 ml albumin-dextrose-catalase (ADC) enrichment (Difco), 2g/L glucose, and 2.5 ml 25% Tween 80. 7H9 CAS medium was prepared by supplementing Middlebrook 7H9 with 4g/L glucose, 0.3 g/L casitone, 0.81 g/L NaCl, and 2.5 ml 25% Tween 80. Glycerol-alanine-salts with iron (GAST/Fe) medium, pH 6.6, was supplemented with 0.3 g/L Bacto Casitone (Difco), 0.05g/L ferric ammonium citrate, 4 g/L dibasic potassium phosphate, 2 g/L citric acid, 1 g/L L-alanine, 1.2 g/L MgCl_2_, 0.6 g/L potassium sulfate, 2 g/L ammonium chloride, 1.8 ml of 10 g/L sodium hydroxide, 10 ml glycerol, and 2.5 ml 25% Tween 80. Cultures were incubated at 37°C in sealed culture flasks with no agitation. Cells were plated onto Middlebrook 7H10 agar plates with 19.47 g/L 7H10 agar base supplemented with 100 ml OADC and 5 ml glycerol. A GFP reporter strain, H37Rv-GFP (Chan et al., 2002; Abrahams et al., 2012), and Mtb bioluminescent reporter strains, P*iniB*-LUX and P*recA*-LUX, were grown in 7H9 OADC with 20 µg/ml kanamycin (Naran et al., 2016). Spontaneous drug resistant mutants were grown in 7H9 OADC without antibiotic selection. Mtb strains with resistance-conferring mutations in *mmpL3*, *dprE1*, *qcrB*, *katG*, or upstream of *inhA* were grown in various media, dependent on the assay, without antibiotic selection. Similarly, the deletion mutants, Δ*tap* and CydKO were grown in 7H9 CAS or 7H9 ADC. *M. smegmatis* was grown in 7H9 OADC media.

### Source of Compounds

The compounds were selected from the National Cancer Institute (NCI) chemical database. A portion of the NCI database is available to the public at https://cactus.nci.nih.gov/ncidb2.2/ and several search functions are available in the NCI open database structures at this website. These compounds were selected from a large pool of compounds available in the database based on computerized substructure searching of TB active pharmacophores. The selection of the pharmacophores was facilitated by the existing NIAID chemical/biological database for HIV and TB at https://chemdb.niaid.nih.gov/. The corresponding NSC numbers for the tested compounds are listed: 127-09 (NSC37381), 127-17 (NSC351686), 127-21 (NSC624736), 127-23 (NSC641601), 127-11 (NSC123112), 127-13 (NSC270389), 127-14 (NSC275424), 127-15 (NSC293875), 127-18 (NSC353903), 127-19 (NSC369060), 127-20 (NSC603173), 127-22 (NSC635076), MN-6925 (NSC155263), and MN-9483 (NSC84486).

### Drug Susceptibility Testing

Compound solutions were prepared as 10 mM stocks in DMSO and stored at −80°C. The MIC for each compound was determined by performing the microbroth dilution assay (Abrahams et al., 2012) and quantitatively analyzed with the colorimetric alamarBlue cell viability reagent (Merck). Briefly, each compound at a starting concentration of 50 µM was diluted two-fold in a clear-well, round-bottom 96-well microtiter plate. Mtb was grown to an OD_600_ of 0.5 (~ 10^8^ cells/ml) and diluted 1,000-fold before adding an equal volume to each well, totaling 100 µl. The plate was sealed and incubated at 37°C for 13 days after which 10 µl of 0.01% alamarBlue was added and incubated for a further 24 h. Fluorescence was recorded using a SpectraMax i3x plate reader (Molecular Devices) at excitation and emission wavelengths of 540 and 590 nm, respectively, and the MIC_90_, the lowest drug concentration to inhibit growth by more than 90%, was determined from the dose-response curve.

### Isolation and Characterization of Spontaneous Drug-Resistant Mutants and Analysis of Mutations

Mutants of Mtb resistant to the compounds under investigation were isolated as previously described (Singh et al., 2017). In brief, ~ 5×10^8^–10^9^ cells/ml from a late logarithmic-phase culture (OD_600_ = 0.8–1.0) was concentrated 100× and 100 µl spread onto 7H10 agar containing compound at 5×, 10×, 20×, or 50× the calculated MIC in liquid culture. Plates were incubated for 3–4 weeks. Individual colonies emerging on the plates were grown in drug-free 7H9 OADC to an OD_600_ of 0.5 and tested for drug susceptibility, as described above, to determine the level of resistance. A small number of individual colonies arose on the plates. For compounds 127-09, 127-17, 127-21, 127-23, 127-13, 127-14, 127-15, 127-18, 127-19, 127-20, 127-22, MN-6925, and MN-9483, the number of colonies picked for drug susceptibility testing were 5, 13, 8, 9, 17, 15, 11, 7, 6, 11, 11, 5, and 7 respectively. No spontaneous resistant mutants (SRMs) could be isolated in the case of compound 127-11. Confirmed mutant clones demonstrating heritable resistance, as determined by a ≥8-fold increase in MIC, were grown to an OD_600_ of 0.5 in 50 ml 7H9 OADC (in the absence of compound) and the cells harvested, and DNA extracted using the cetyltrimethylammonium bromide (CTAB) method in preparation for whole genome sequencing (WGS). Libraries were prepared using the TruSeq kit following the manufacturers protocol and sequenced on the Illumina NovaSeq at the Texas A&M University Genomics and Bioinformatics Service. Reads with an average read depth of 360-fold were mapped to the reference genome, Mtb H37RvMA (GenBank accession NZ_CM002884.1) using BWA (Li and Durbin, 2009) and polymorphisms (single nucleotide polymorphisms (SNPs) and insertions/deletions) were identified using variant-calling scripts developed in-house. PE_PGRS genes were excluded from the analysis. The sequencing data have been deposited in NCBI SRA under BioProject accession number PRJNA645974. The location of mutated residues in the respective proteins were analyzed using the protein modeling software Protein Homology/analogY Recognition Engine V2.0 (Phyre2) (Kelley et al., 2015).

### Mammalian Cell Cytotoxicity Assay

Compounds were screened for cytotoxicity against the Vero cell line (kidney epithelial cells) using the 3-(4,5-dimethylthiazol-2-yl)-2,5-diphenyltetrazoliumbromide (MTT)-assay as previously described (Wilson et al., 2017). IC_50_ values were determined from the dose-response curves. The selectivity index (SI) of each compound was calculated by dividing the IC_50_ value by the MIC value.

### GFP Release Assay

The assay was conducted as described previously (Kumar et al., 2012; Park et al., 2017). Briefly, H37Rv-GFP was grown to an OD_600_ of 0.2–0.3 in 7H9 OADC and exposed to compound at 1× or 10×MIC. Meropenem was used as the lytic drug control and linezolid as the non-lytic control. Every 24 h over a period of 6 days, 200 µl of culture was harvested and pelleted by centrifugation. Approximately 200 µl of the supernatant was transferred to a black, clear-bottom 96-well microtiter plate (Greiner CellStar^®^) and fluorescence (excitation 540 nm; emission 590 nm) measured using the SpectraMax i3x plate reader (Molecular Devices). Fluorescence intensity was normalized to the drug-free control.

### Bioluminescence Assay

The assay was performed as previously described (Naran et al., 2016). Briefly, the P*iniB*-LUX or P*recA*-LUX reporter strains of Mtb were grown to an OD_600_ ~ 0.4 and diluted to an OD_600_ ~ 0.04 in 7H9 OADC. The cells were inoculated into white, clear bottom 96-well microtiter plates (Greiner CellStar^®^) containing a two-fold serial dilution of each compound, prepared as described for the drug susceptibility testing assay. The plates were incubated at 37°C and the luminescence recorded every 24 h for 9 days using a SpectraMax i3x plate reader (Molecular Devices). The readings were plotted over time. Isoniazid (INH) and ciprofloxacin (CIP) were included as cell-wall-acting and genotoxic drug controls, respectively (Naran et al., 2016).

## Results

### Characterization of the Anti-TB Activity Profiles of Compounds Selected From a Pharmacophore-Based Virtual Screen

**Figure 1:**
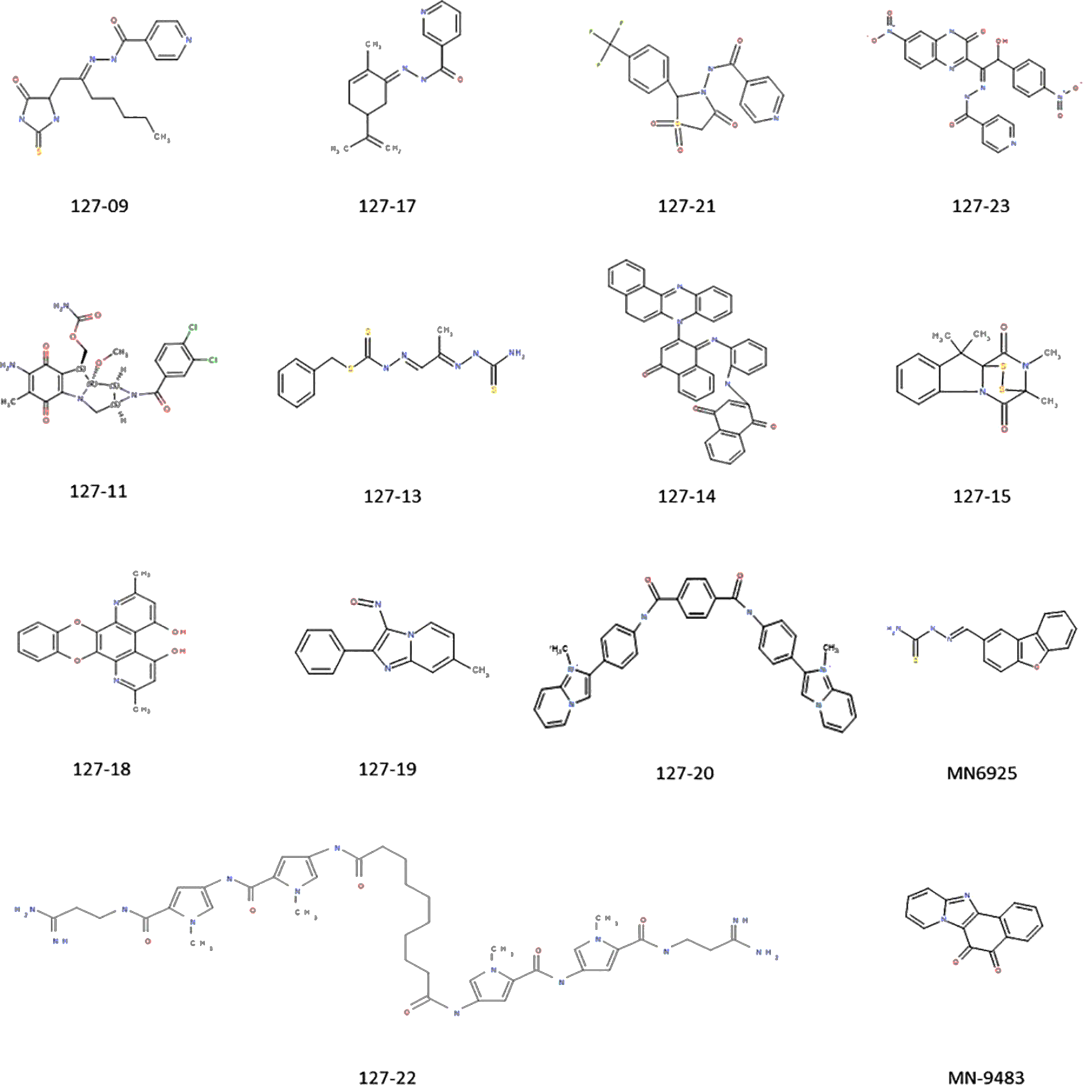
Compounds exhibiting activity against H37Rv.

A set of 14 compounds, which were selected from the NCI database using a TB active pharmacophore substructure search, were obtained from the Division of AIDS at the NIAID. Three of the compounds share a pyridine-4-carboxamide motif (127-09, 127-21, and 127-23) and another contains a pyridine-3-carboxamide motif (127-17) with all four carrying a hydrazide, analogous to INH. One is structurally related to mitomycin C (127-11), and the other nine are structurally diverse (**Figure 1**). The compounds were tested for activity against replicating Mtb H37Rv in different media (**Table 1**) as specific carbon sources and/or media components are known to influence inhibitory activity of antimycobacterial agents (Franzblau et al., 2012). The 10 most active compounds had MIC values ranging from 0.4–3.1 µM in 7H9 OADC whereas the remaining four were less potent (MICs = 6.3–12.5 µM). Thirteen of the compounds showed ~5-fold increased potency in GAST/Fe (protein-free) media; the only outlier was 127-13, which showed an eight-fold lower activity in GAST/Fe. Otherwise, only minor (<4-fold) differences in MIC values were observed across the range of media tested. Compounds 127–11, 127–19, 127–20, and 127–22 also showed reasonably potent activity against *Mycobacterium smegmatis*, demonstrating MICs in the range of 0.4–6.3 µM (**Supplementary Table 2**). All compounds, with the exception of 127-18 had a selectivity index (SI) above 10, indicative of effective antimycobacterial activity and (comparatively) low cytotoxicity against the mammalian Vero cell line (**Table 1**) (Orme, 2001). Compounds 127-20, 127-17, 127-22 and MN-9483 exhibited the highest SI values (100 – >3000).

**Table 1:** Inhibitory activity against replicating Mtb and mammalian cell toxicity of compounds.

Compound identification	Compound name	Other identifier	Log P	MIC (µM) in supplemented media				Inhibition and selectivity (Vero cell line)	
				7H9 OADC	7H9 ADC	7H9 CAS	GAST/Fe	Cyto-toxicity IC_50_ (µM)	SI (IC_50_/MIC)
127-09	N-[(E)-1-[(5-oxo-2-thioxo-imidazolidin-4-yl)methyl]hexylideneamino]pyridine-4-carboxamide	Pyridine carboxamide analogue	2.18	1.6–3.1	1.6	0.4	0.4	–	–
127-17	N-[(E)-(5-isopropenyl-2-methyl-cyclohex-2-en-1-ylidene)amino]pyridine-3-carboxamide	Pyridine carboxamide analogue	2.94	1.6–3.1	6.3	1.6	1.0	420	280
127-21	N-[1,1,4-trioxo-2-[4-(trifluoromethyl)phenyl]-1,3-thiazolidin-3-yl]pyridine-4-carboxamide	Pyridine carboxamide analogue	1.72	1.6–3.1	6.3	1.6	0.5	> 160	> 25.4
127-23	N-[(E)-[2-hydroxy-1-(6-nitro-3-oxo-4H-quinoxalin-2-yl)-2-(4-nitrophenyl)ethylidene]amino]pyridine-4-carboxamide	Pyridine carboxamide analogue	3.74	3.1–6.3	12.5	3.1	3.1	> 130	> 10.4
127-11	[amino-(3,4-dichlorobenzoyl)-methoxy-methyl-dioxo-yl]methyl carbamate	Mitomycin derivative T 56	1.03	0.4	0.1	0.8	< 0.2	3.75	62.5
127-13	benzyl N-[(E)-[(2E)-2-(carbamothioylhydrazono)propylidene]amino]carbamodithioate		3.42	1.6–3.1	1.6	1.6	25	38.4	64
127-14	2-[2-[(E)-[2-(5H-benzo[a]phenazin-7-yl)-4-oxo-1-naphthylidene]amino]anilino]naphthalene-1,4-dione		5.45	12.5	6.3	6.3	6.3	98.5	15.6
127-15	10H-3,10a-Epidithiopyrazino[1,2-a]indole-1,4-dione, 2,3-dihydro-2,3,10,10-tetramethyl-		4.00	0.4–0.8	0.1	0.4	< 0.2	19	31.6
127-18	2,7-dimethyl-[1,4]benzodioxino[2,3-f][4,7]phenanthroline-4,5-diol		2.81	12.5	25	25	1.4	163	6.52
127-19	7-methyl-3-nitroso-2-phenyl-imidazo[1,2-a]pyridine		3.11	0.8–1.6	0.7	0.1	< 0.2	52.7	33
127-20	N1,N4-bis[4-(1-methylimidazo[1,2-a]pyridin-1-ium-2-yl)phenyl]terephthalamide		5.80	1.6	1.4	–	< 0.2	> 4300	> 3071
127-22	N,N’-bis[5-[[5-[(3-amino-3-imino-propyl)carbamoyl]-1-methyl-pyrrol-3-yl]carbamoyl]-1-methyl-pyrrol-3-yl]decanediamide		−2.06	3.1	1.6	12.5	< 0.2	54.22	100
MN-6925	[(E)-dibenzofuran-2-ylmethyleneamino]thiourea		2.69	6.3–12.5	3.1	6.3	3.1	74	> 23.9
MN-9483	Naphtho[1’,2’:4,5]imidazo[1,2-a]pyridine-5,6-dione		1.71	3.1	1.6	3.1	0.4	> 252	> 158

Is not determined.

Drug-induced lytic activity was monitored by measuring the release of GFP from H37Rv-GFP treated with compound at 1×MIC or 10×MICs as described previously (Kumar et al., 2012; Park et al., 2017). Meropenem which induces rapid lysis of Mtb by inhibiting the cell wall remodeling enzymes, D,D-carboxypeptidase and L,D-transpeptidase was used as a lytic control whereas the bacteriostatic translation inhibitor, linezolid (Zhang et al., 2014), was used as a non-lytic control (**Figure 3**). Meropenem, in both the 1×MIC and 10×MIC experiments, led to a steady increase in GFP fluorescence signal over time, whereas linezolid produced no fluorescence signal other than on day 6 in the 10×MIC experiment, when a weak signal was detected. Compound 127-18 was intrinsically fluorescent, yielding a drug-concentration-dependent, false positive signal in this assay. Furthermore, all compounds produced a late fluorescence signal at day 6 in the 10×MIC experiment, presumably attributable to cell death. Compound 127-11 led to GFP release from Mtb at both drug concentrations indicative of true lytic activity, albeit with markedly slower kinetics than the meropenem control. Of the putative cell wall targeting agents, none produced a significant fluorescence signal. Although INH and the pyridine carboxamides target the cell wall, none elicited a signal in the GFP release assay. One possible explanation is that the disruption in cellular metabolism due to inhibition of InhA activity may block growth earlier than the disruption in cell wall integrity, which is required for GFP release to occur.

### Broad Mechanistic Characterization Using Reporter Strains

Reporter strains of Mtb have been developed that enable rapid classification of antimycobacterial agents into three broad mechanistic classes: compounds that act on the cell wall, compounds that interfere with DNA metabolism, and inhibitors of mycobacterial energy metabolism. Another mutant strain has been developed to establish whether inhibitors are subject to efflux. These were used to screen the compounds.

**Figure 2:**
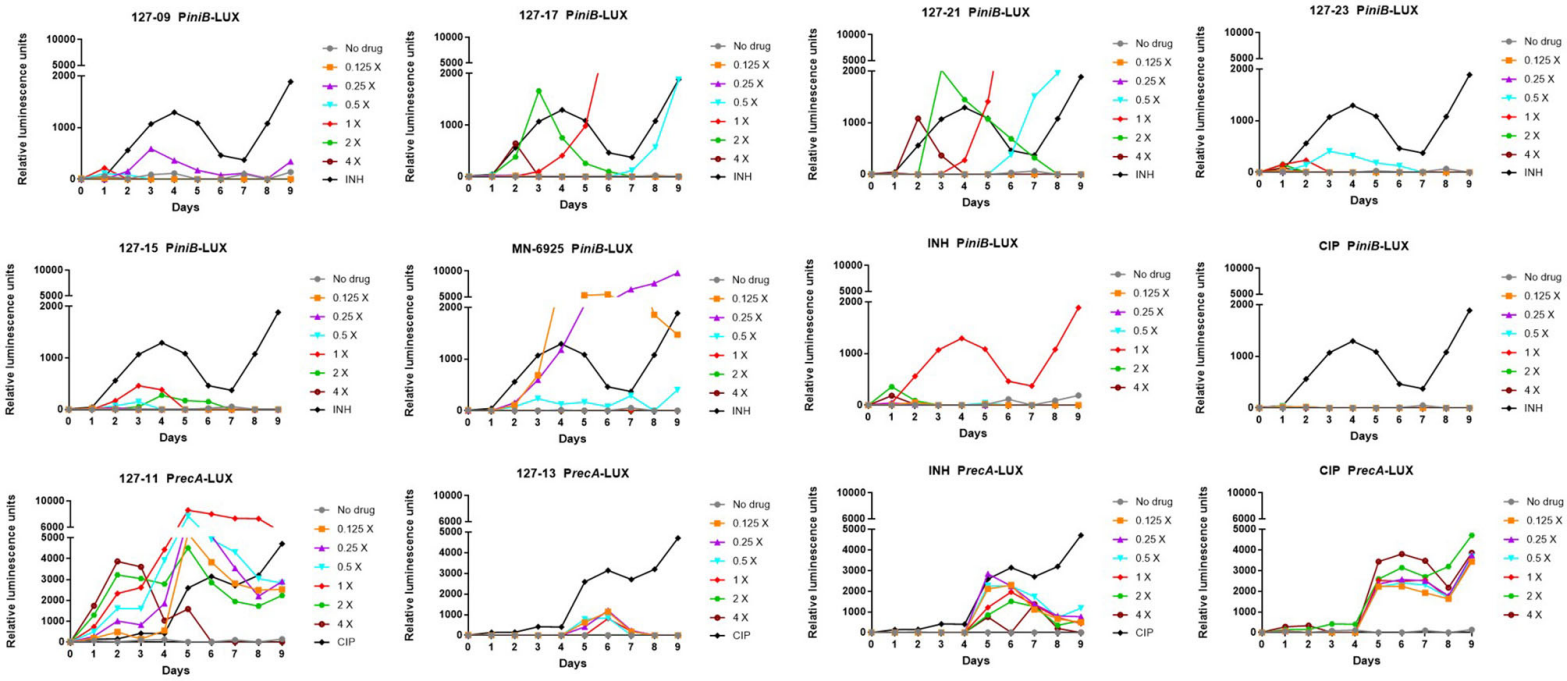
Induction of *iniBAC* or *recA* bioluminescent reporter in response to antimycobacterial agents with unknown MoAs at concentrations ranging between 0.125-4× MIC, monitored over 9 days. For P*iniB*-LUX, INH was used as the positive control at a concentration of 0.39 µM (1× MIC); CIP was used as the negative control. The *iniBAC* response is shown for 127-09, 127-17, 127-21, 127-23, 127-13, 127-15, MN-6925, and the controls. For P*recA*-LUX, CIP was used as the positive drug control at 3.13 µM (2× MIC), although concentrations in the range of 0.2–6.25 µM (0.125-4× MIC) all gave similar luminescence profiles; INH was the negative control. The *recA* response is shown for 127-11, 127-13 and the controls. RLU, relative luminescence unit.

A luminescence-based reporter assay (Naran et al., 2016) developed to detect cell wall stress or DNA damage induced by exposure of Mtb to antimycobacterial agents was used to establish which of the compounds fall into either of these broad mechanistic categories. This assay is reliant on the transcriptional responsiveness of the *iniBAC* promoter to cell wall stress (Alland et al., 2000) and the *recA* promoter to genotoxic stress (Brooks et al., 2001), and uses a luminescence-based readout. INH and CIP were included in all assays as positive controls for induction of *iniBAC* and *recA* induction, respectively. All compounds were tested in these assays at drug concentrations ranging from 0.125×MIC to 4×MIC. Consistent with previous observations, INH triggered a luminescence signal in P*iniB*-LUX peaking at 1×MIC (Naran et al., 2016), and also elicited a late, weak signal in P*recA*-LUX at all concentrations Conversely, CIP generated a strong signal in P*recA*-LUX, peaking at 0.125x−4xMIC, but elicited no response in P*iniB*-LUX at any of the concentrations tested. Six of the compounds elicited a positive *iniBAC* response, suggesting that they interfere with cell envelope biogenesis (**Figure 2**; **Supplementary Table 3**). Compounds 127–09 and 127–23 elicited low-level signals, 3–5-fold lower than the INH control, peaking at day 3 at sub-inhibitory concentrations (0.25× and 0.5×MIC, respectively). 127-15 also induced a low-level signal in P*iniB*-LUX, peaking at day 3 at 1×MIC whereas 127-17 and 127-21 triggered responses 2-3-fold higher than INH at 2×MIC, also peaking at day 3. MN-6925 elicited the most distinctive response, reaching a level five-fold higher than the INH control and showing the strongest signal at sub-inhibitory concentrations of compound.

To establish whether the compounds identified from the P*iniB*-LUX assay as putative “cell wall actives” act on two promiscuous cell envelope targets, DprE1 and MmpL3, they were tested in secondary screens against Mtb strains carrying known resistance-conferring mutations in *dprE1* or *mmpL3* (**Supplementary Table 4**). The DprE1^Y314C^, DprE1^Y314H^, DprE1^P116S^, and MmpL3^G253E^ mutants were tested in two media types. At least 15 inhibitors have been described that target DprE1 (Degiacomi et al., 2020). Resistance to one of these compounds, TCA1, maps to Y314C (Wang et al., 2013), a mutation which causes a structural change in the enzyme active site (Liu et al., 2017). Resistance to N-alkyl-5-hydroxypyrimidinone carboxamides was associated with the amino acid substitutions Y314C, Y314H, and P116S in DprE1 (Oh et al., 2018). Likewise, the G253E mutation in MmpL3 has been implicated in resistance to a wide range of MmpL3 inhibitors which include the tetrahydropyrazolopyrimidine THPP1, the 1,2-diamine SQ109, the adamantyl urea, AU1235, the indolecarboxamides NITD-304 and NITD-349 (Li et al., 2019), and other diverse inhibitors (Williams et al., 2019). None of the six compounds designated as cell wall active based on the P*iniB*-LUX assay showed cross-resistance to the DprE1 or MmpL3 mutants. Interestingly, however, MN-6925 showed increased potency against all four DprE1 and MmpL3 mutants, but only in 7H9 ADC media.

**Figure 3:**
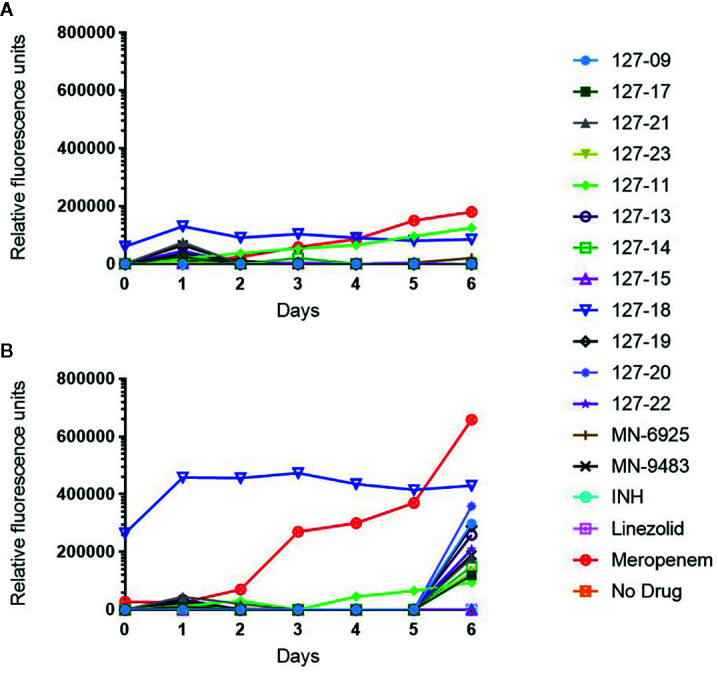
Measurement of released GFP in culture supernatant following exposure of H37Rv*-*GFP strain to 1× **(A)** or 10× **(B)** MIC of the respective compound. Note 127-18 is an intrinsically fluorescent compound.

A strong sustained *recA* signal was triggered in response to compound 127-11 (**Figure 2**; **Supplementary Table 3**). There was a clear concentration-dependent induction over the range of 0.125×–2×MIC, above which there was a drop in the luminescence signal, presumably due to cell death (**Figure 3**). 127-11 is structurally related to mitomycin C, suggesting that this compound is a DNA crosslinking agent (Tomasz, 1995). The remaining compounds elicited a weak, late signal (around day 5) analogous to INH (**Figure 2**; **Supplementary Table 3**), indicative of indirect DNA damage as a result of drug action, as suggested previously (Naran et al., 2016).

To ascertain whether any of the compounds acted on QcrB, a promiscuous target in mycobacterial energy metabolism, they were tested for hypersensitivity against the CydKO mutant of Mtb in which the cytochrome *bd* oxidase is functionally disrupted (Arora et al., 2014; Moosa et al., 2017). None of the compounds showed differential activity in this mutant compared to wildtype Mtb (**Supplementary Table 4**). To determine whether any of the compounds was a substrate for efflux, they were tested against a knockout mutant in the efflux pump, Rv1258c (Tap) (Ramon-Garcia et al., 2012). Only MN-6925 showed an eight-fold reduction in MIC against the Δ*tap* mutant; however, this potentiating effect was observed exclusively in 7H9 ADC media (**Supplementary Table 4**).

### Mechanistic Insights From Spontaneous Drug-Resistant Mutants

To elucidate mechanisms of action, Mtb was plated onto increasing concentrations of compound (5-50×MIC) to select for SRMs as described under *Materials and Methods* (**Table 2**). SRMs displaying heritable resistance against 127-13, 127-15, 127-17, 127-21, and 127-23 were isolated at frequencies in the range of 10^−6^–10^−7^ (**Table 2**). Two SRMs for each compound were chosen for WGS. WGS analysis of clones with high-level resistance against 127-17, 127-21, and 127-23 were all found to carry mutations in *katG* (**Table 3**). Furthermore, cross resistance to these three compounds as well as 127-09 was observed in Mtb strains carrying the KatG^S315T^ or *inhA* ^−15C→T^ mutations commonly observed in INH-resistant clinical isolates (Lempens et al., 2018). These results indicate, that all four are INH-like prodrugs, which are activated by KatG to target InhA (**Table 4**). The S315T and S315R mutations commonly associated with INH resistance were found in mutants raised against compound 127-21; in contrast compounds 127-17 and 127-23 appeared to interact with KatG in a manner distinct from INH, as evidenced by the identification of the V544F, G285V, and *741R mutations in SRMs to these compounds, which have not been associated previously with INH resistance (**Table 3**). The G285 residue is located on the surface at the mouth of the tunnel in relation to the active site heme, not far from S315, whereas V544 is located distal to this, buried within the C-terminal domain of KatG. The *741R mutation at the stop codon of *katG* is predicted to extend the protein product by creating a C-terminal fusion with the open reading frame of Rv1907c. The conclusion that 127-21, 127-17, and 127-23 interact with KatG differently from INH was corroborated by screening these mutants for cross-resistance to INH: while the KatG^S315R^ mutation conferred cross-resistance to INH, the KatG*^741R^ and KatG^G285V^ mutations associated with resistance to 127-17 and 127-23, respectively, had no effect on the susceptibility of Mtb to INH. The KatG mutants that were sensitive to INH also retained sensitivity to 127-09 but were cross-resistant to the other pyridine carboxamides (**Table 4**).

**Table 2:** Drug resistant mutants isolated against selected compounds.

Compound	MIC against H37Rv (µM)	MIC against SRM (µM)	Fold shift in SRM MIC (over H37Rv)	Mutation frequency
127-17^R^ SRM-1	1.6–3.1	50	31×	2.0×10^−7^
127-21^R^ SRM-1	1.6–3.1	50	31×	2.0×10^−7^
127-21^R^ SRM-2	1.6–3.1	50	31×	2.0×10^−7^
127-23^R^ SRM-1	3.1–6.3	50	16×	1.5×10^−7^
127-23^R^ SRM-2	3.1–6.3	25	8×	1.5×10^−7^
127-13^R^ SRM-1 127-13^R^ SRM-2	1.6–3.1	12.5	8×	9.0×10^−6^
127-15^R^ SRM-1 127-15^R^ SRM-2	0.4–0.8	6.3	8×	2.5×10^−6^

MICs in 7H9 OADC media.

**Table 3:** Mutations identified by WGS of Mtb mutants spontaneously resistant to selected compounds.

Compound	Gene	Mutation/s	Product
127-17^R^ SRM-1	*katG* *fadD26*	*741R G74*	Catalase-peroxidase-peroxynitritase Fatty-acid-AMP ligase
127-21^R^ SRM-1	*katG* *ppsC* Rv3755c	Y597N; S315N^a^ Q1085* D100G	Catalase-peroxidase-peroxynitritase Phenolpthiocerol synthesis type-I polyketide synthase Unknown (conserved protein)
127-21^R^ SRM-2	*katG*	S315R	Catalase-peroxidase-peroxynitritase
127-23^R^ SRM-1	*katG* *ppsB*	V544F Q945P	Catalase-peroxidase-peroxynitritase Phenolpthiocerol synthesis type-I polyketide synthase
127-23^R^ SRM-2	*katG* *ppsC* *phoP*	G285V +c insertion at nt 680 IS6110 insertion at nt 210	Catalase-peroxidase-peroxynitritase Phenolphthiocerol synthesis type-I polyketide synthase Possible two component system response transcriptional positive regulator PhoP
127-13^R^ SRM-1 127-13^R^ SRM-2	Rv0678 *fadD26* Rv3479	-g deletion at nt 198 S553* N831K	Regulator of MmpS5-MmpL5 efflux pump Fatty-acid-AMP ligase Transmembrane protein
127-15^R^ SRM-1 127-15^R^ SRM-2	Rv2466c *fadD26*	-t deletion at nt 151 G74*	Mycothiol dependent reductase, D Fatty-acid-AMP ligase

*stop codon; nt, nucleotide.

^a^Since these sites were heterogeneous, 127-21^R^ SRM-1 was probably a mixture of 2 mutants, each with a different KatG mutation.

**Table 4:** Evaluation of cross resistance of pyridine carboxamide analogues against KatG^S315T^, *inhA* ^−15C→T^ and spontaneous drug resistant mutants.

Mtb strain	MIC (µM)					
	H37Rv	KatG^S315T^	*inhA* ^−15C→T^	127-17^R^ SRM-1	127-21^R^ SRM-2	127-23^R^ SRM-2
**Compound**						
127-09	1.6-3.1	25	6.3	6.3	12.5	3.1
127-17	1.6-3.1	50	25	50	50	50
127-21	1.6-3.1	50	12.5	25	50	50
127-23	3.1-6.3	50	25	50	50	50
INH	0.1	1.6	0.4	0.1	6.3	0.1

Tested against 127-17^R^ SRM-1 *^741R^, 127-21^R^ SRM-2 ^S315R^ and 127-23^R^ SRM ^G285V^.

SRMs displaying intermediate-level resistance (~8-fold higher MIC than wildtype) were also isolated against compounds 127-13 and 127-15 (**Table 2**). WGS analysis of two SRMs to 127-13 identified a frameshift mutation in Rv0678, the regulator of the MmpS5-MmpL5 efflux pump, whereas two SRMs to 127-15 carried a frameshift mutation in *dsbA*, which encodes a mycothiol dependent reductase (**Table 3**). In addition to these probable resistance-conferring mutations, all SRMs sequenced in this study also carried mutations in genes involved in PDIM biosynthesis (*fadD26*, *ppsB, ppsC*). Mutations in the PDIM locus which affect the ability to produce this abundant virulence lipid are commonly observed when Mtb is passaged *in vitro* and are unlikely to be related to the MoAs of these compounds (Domenech and Reed, 2009). Finally, unlike the six compounds described above, SRMs could not be isolated on solid media for the remaining eight compounds under any of the conditions tested.

## Discussion

In this study, we describe the application of a set of phenotypic assays to elucidate the MoAs of 14 compounds with activity against replicating Mtb in the 0.1–25 µM range and selectivity indices ranging from 6 to >3,000. A SciFinder (Scifinder, 2020) search found an exact match to 127-09, which had been reported more than six decades ago as a compound that displayed good activity against Mtb and was less toxic than INH in a mouse model (Archer et al., 1956). To the best of our knowledge, the 13 other compounds have not been previously reported or characterized in terms of antitubercular activity. Among the most potent compounds, 127-11, demonstrated genotoxic and lytic activity against Mtb, consistent with its structural similarity to mitomycin C, a drug commonly used for the treatment of certain cancers, with known activity against growing and hypoxic cultures of *Mycobacterium bovis* (Peh et al., 2001) and efficacy against other bacterial persisters (Kwan et al., 2015). However, supporting a non-specific MoA involving DNA cross-linking, 127-11 displayed significant mammalian cell toxicity and hence, a modest SI.

Six of the compounds were classified as cell wall active based on responsiveness of the *iniBAC* promoter upon exposure of Mtb to these compounds. Of the six, three (127-09, 127-21, and 127-23) contained a pyridine-4-carboxamide motif shared with INH, and another (127-17) contained a related pyridine-3-carboxamide motif. All four compounds share a common MoA with INH, as inferred from WGS of spontaneous resistant mutants and/or cross-screening against INH-resistant mutants carrying the common resistance-conferring mutations, KatG^S315T^ and *inhA* ^−15C→T^. Therefore, these compounds are most likely prodrugs that are biotransformed by KatG to metabolites that inhibit InhA. However, the KatG*^741R^ mutation selected by resistance to 127-17, and KatG^G285V^ mutation selected by resistance to 127-23 did not confer resistance to INH. This lack of cross-resistance is most likely attributable to the involvement of additional residues in binding these bulky INH analogues to the KatG active site. In a recent study, a 2-pyridone bicyclic scaffold compound was shown to re-sensitize an INH resistant mutant to INH by countering oxidative stress tolerance (Flentie et al., 2019). It would be interesting to establish whether this compound could also restore sensitivity of *katG* mutants to these pyridine carboxamide analogues. It would likewise be useful to assess whether the compounds differ from INH in terms of activity in models of slow- or non-replicating persistence induced by low oxygen, mild hypoxia (Cho et al., 2015; Zheng et al., 2017), carbon starvation (Grant et al., 2013), or a combination of multiple stresses (Deb et al., 2009; Gold et al., 2015). Extensive effort has been made to improve the antitubercular potency while lowering the cytotoxicity of INH, albeit with limited success (Martins et al., 2014; Oliveira et al., 2017; Dragostin et al., 2019). Consistent with this, the pyridine carboxamides investigated here are significantly less potent against Mtb and have lower SI values than INH (SI>2941 based on Vero cell line toxicity) (Phillips et al., 2012).

Displaying a sub-micromolar MIC, 127-15 was one of the most potent compounds investigated in this study. It was designated as cell wall active based on early induction of the *iniBAC* promoter; however, the signal was low and not sustained, perhaps as a result of rapid cell death. Moreover, this compound showed no cross-resistance to the four DprE1 or MmpL3 mutants tested arguing against the involvement of these promiscuous targets in the MoA of 127-15. In contrast, MN-6925 induced a very strong *iniBAC* signal unambiguously identifying it as an inhibitor of cell envelope biogenesis. Interestingly, MN-6925 was distinguished by the fact that it was the only compound that showed increased potency against the MmpL3^G235E^, DprE1^Y314H^, DprE1^Y314C^, DprE1^P116S^, and Δ*tap* mutants in 7H9 ADC media. While the mechanistic basis of this observation is unclear, it is worth noting that mutations in MmpL3 can result in alterations in the hydrophobicity of the mycobacterial cell wall (McNeil et al., 2017) and thereby potentially increase compound permeability, and that disruption of Tap results in generalized repression of genes involved in cell wall biosynthesis during stationary phase (Ramon-Garcia et al., 2012).

A deletion in Rv0678, a negative regulator of the MmpL5-MmpS5 efflux system led to intermediate-level resistance to compound 127-13. Mutations in this MarR-like regulator were initially identified in azole-resistant mutants of Mtb (Milano et al., 2009) and then in clofazimine- as well as bedaquiline-resistant mutants (Andries et al., 2014; Hartkoorn et al., 2014). Mutations in this regulator have been found to confer resistance to other chemotypes (Ioerger et al., 2013), consistent with non-specific compound efflux *via* induction of the MmpL5-MmpS5 system as a generalized mechanism of resistance in Mtb.

An eight-fold increase in MIC was also observed for drug resistant mutants isolated against compound 127-15. This was associated with a loss-of-function mutation in Rv2466c, encoding a mycothiol-dependent reductase, DsbA, which is essential for Mtb survival under oxidative stress (Albesa-Jové et al., 2014; Rosado et al., 2017). This enzyme has been implicated in the activation of the prodrug TP053, a thienopyrimidine derivative, by a dithiol-disulfide mechanism (Albesa-Jové et al., 2014). The compound 127-15 is therefore likely to be metabolized by Rv2466c by reduction of the disulfide bond to generate an active metabolite that could inhibit multiple cellular targets in Mtb.

In summary, this study has demonstrated the utility of reporter strains of Mtb for rapidly classifying phenotypic screening hits into broad mechanistic categories. This approach provides a means of judiciously applying scarce resource to hit expansion or hit-to-lead activities while gaining further mechanistic insight into the most promising hits. Of the 14 compounds investigated in this study, five were found to be prodrugs that require activation *via* mechanisms described previously for other prodrugs, including INH. These were readily identified by sequencing of resistant mutants that arose at comparatively high frequencies. This finding is consistent with the over-representation of prodrugs among new and existing TB drugs, and further underscores Mtb-mediated biotransformation as a prominent feature of anti-tubercular drug MoAs (Awasthi and Freundlich, 2017). Half of the compounds proved refractory to MoA elucidation using the assays applied here. Therefore, while phenotypic screening provides a convenient means of identifying new inhibitory scaffolds, MoA elucidation can remain a challenging undertaking (Singh and Mizrahi, 2017). It will be interesting to determine whether high-throughput chemical-genetic approaches, such as the PROSPECT system for screening hypomorph libraries (Johnson et al., 2019), could be informative in such cases.

## Data Availability Statement

The sequencing data have been deposited in NCBI SRA under BioProject accession number PRJNA645974.

## Author Contributions

VM, DW, and MC conceived and designed the experiments. MN provided the compounds, SF performed MIC and cytotoxicity assays, AJ and RS performed MIC assays, TI performed the WGS analyses, and MC performed all other experiments. MC and VM wrote the manuscript. All authors contributed to the article and approved the submitted version.

## Funding

This work was funded by grants from the South African Medical Research Council (to VM), the Strategic Health Innovations Partnerships (SHIP) Unit of the SAMRC (to DW and VM), the National Research Foundation of South Africa (to VM), the Oppenheimer Memorial Trust (to VM), the Bill & Melinda Gates Foundation (OPP1158806, *via* subaward from the FNIH) and an Early-Career Researcher Award from the University of Cape Town (to MC).

## Conflict of Interest

The authors declare that the research was conducted in the absence of any commercial or financial relationships that could be construed as a potential conflict of interest.
