# Supplementary material for: Biological Profiling Enables Rapid Mechanistic Classification of Phenotypic Screening Hits and Identification of KatG Activation-Dependent Pyridine Carboxamide Prodrugs With Activity Against Mycobacterium tuberculosis
Source: Front Cell Infect Microbiol. 2020 Nov 13;10:582416. doi: 10.3389/fcimb.2020.582416 (PMC7691319; doi:10.3389/fcimb.2020.582416)
Supplement: Supplementary file 1 [file Table_1.docx]

Supplementary Material

**Supplementary Table 1.** Bacterial strains used in this study

| **Strains** | **Description** | **Reference/ source** |
| --- | --- | --- |
| *M. smegmatis* | Strain mc^2^155, high frequency transformation mutant of *M. smegmatis* mc^2^6; ATCC 706 | (Snapper et al., 1990) |
| H37Rv | *M. tuberculosis* strain H37RvMA isolate ATCC no. 27294, virulent laboratory strain | Dr. Christopher Sassetti  (Ioerger et al., 2010) |
| H37Rv-GFP | H37Rv derivative harboring a vector carrying GFP expressed from the pMSP12 promoter | (Chan et al., 2002;Abrahams et al., 2012) |
| Mtb-P*iniB*-LUX | H37Rv::pMV306 carrying LUXG13 expressed from the *iniB* promoter | (Naran et al., 2016) |
| Mtb-P*recA*-LUX | H37Rv::pMV306 carrying LUXG13 expressed from the *recA* promoter | (Naran et al., 2016) |
| Mtb-MmpL3^G253E^ | H37Rv derivative carrying resistance-conferring mutation G253E in MmpL3 | Dr. Vinayak Singh |
| Mtb-DprE1^Y314H^ | H37Rv derivative carrying resistance-conferring mutation Y314H in DprE1 | (Oh et al., 2018) |
| Mtb-DprE1^P116S^ | H37Rv derivative carrying resistance-conferring mutation P116S in DprE1 | (Oh et al., 2018) |
| Mtb-DprE1^Y314C^ | H37Rv derivative carrying resistance-conferring mutation Y314C in DprE1 | (Oh et al., 2018) |
| CydKO | H37Rv derivative in which *cydC* is insertionally inactivated by *aph* | (Arora et al., 2014;Moosa et al., 2017) |
| CydKO-QcrB^A317T^ | H37Rv *cydC::aph* derivative carrying A317T mutation in QcrB | (Arora et al., 2014) |
| Δ*tap* | H37Rv derivative Rv1258 (*tap*) deletion mutant | Dr. Jose A. Aínsa |
| Mtb-KatG^S315T^ | Mtb strain resistant to INH | (Torres et al., 2015) |
| Mtb-inhA^-15C→T^ | Mtb strain resistant to INH | (Torres et al., 2015) |
| 127-17^R^ SRM-1^a^ | H37Rv derivative carrying resistance-conferring mutation *741R in KatG | This study |
| 127-21^R^ SRM-1 ^a^ | H37Rv derivative carrying resistance-conferring mutations Y597N and S315N in KatG | This study |
| 127-21^R^ SRM-2 ^a^ | H37Rv derivative carrying resistance-conferring mutation S315R in KatG | This study |
| 127-23^R^ SRM-1 ^a^ | H37Rv derivative carrying resistance-conferring mutation V544F in KatG | This study |
| 127-23^R^ SRM-2 ^a^ | H37Rv derivative carrying resistance-conferring mutation in G285V in KatG | This study |
| 127-13^R^ SRM-1 ^a^  127-13^R^ SRM-2 ^a^ | H37Rv derivative carrying a -g deletion mutation at nt 198 in Rv0678 | This study |
| 127-15^R^ SRM-1 ^a^  127-15^R^ SRM-2 ^a^ | H37Rv derivative carrying a -t deletion at nt 151 in Rv2466c | This study |

^a^ Additional non-resistance conferring mutations in SRMs are listed in Table 3

^b^ * denotes a stop codon

**Supplementary Table 2.** Activity of compounds against *M. smegmatis*^a^

| **Compound** | **Compound name** | **MIC (µM) (7H9 OADC)** |
| --- | --- | --- |
| 127-11 | [amino-(3,4-dichlorobenzoyl)-methoxy-methyl-dioxo-yl]methyl carbamate | 3.1 |
| 127-19 | 7-methyl-3-nitroso-2-phenyl-imidazo[1,2-a]pyridine | 6.3 |
| 127-20 | N1,N4-bis[4-(1-methylimidazo[1,2-a]pyridin-1-ium-  2-yl)phenyl]terephthalamide | 0.4 |
| 127-22 | N,N'-bis[5-[[5-[(3-amino-3-imino-propyl)carbamoyl]-1-methyl-pyrrol-3-yl]carbamoyl]-1-methyl-pyrrol-3-yl]  Decanediamide | 6.3 |

^a^The remaining 10 compounds not listed here showed weak activity against *M. smegmatis* (MIC > 50 µM) and are therefore not listed here.

**Supplementary Table 3 (related to Figure 2).** Cell wall stress and DNA damage response profiles of compounds assessed using bioluminescent reporter strains Mtb-P*ini*B-LUX and Mtb-P*recA*-LUX^a^

|  | ***iniBAC* response** | | ***recA* response** | |
| --- | --- | --- | --- | --- |
| **Compound** | **P*iniB*-LUX** | **Assessment** | **P*recA*-LUX** | **Assessment** |
| 127-09 | 🗸 | Early sustained | × | Late indirect |
| 127-17 | 🗸 | Early sustained | × | Late indirect |
| 127-21 | 🗸 | Early sustained | × | Late indirect |
| 127-23 | 🗸 | Early sustained | × | Late indirect |
| 127-11 | × | No response | 🗸 | Early sustained |
| 127-13 | × | No response | × | Late indirect |
| 127-14 | × | No response | × | Late indirect |
| 127-15 | 🗸 | Early | × | Late indirect |
| 127-18 | × | No response | × | Late indirect |
| 127-19 | × | No response | × | Late indirect |
| 127-20 | × | No response | × | Late indirect |
| 127-22 | × | No response | × | Late indirect |
| MN-6925 | 🗸 | Early sustained | × | Late indirect |
| MN-9483 | × | No response | × | Late indirect |
| INH | 🗸 | Early sustained | × | Late indirect |
| CIP | × | No response | 🗸 | Early sustained |

^a^The profiles for selected compounds are represented in Figure 2. INH served as the positive control in P*iniB*-LUX and CIP as the negative control. Conversely, CIP served as the positive control in P*recA*-LUX and INH as the negative control.

**Supplementary Table 4. Counter-screening against *M. tuberculosis* strains carrying resistance-conferring mutations in promiscuous targets and other informative mutant strains**^a^

|  | MIC (µM)  7H9 CAS | | | | | | MIC (µM)  7H9 ADC | | | | | | |
| --- | --- | --- | --- | --- | --- | --- | --- | --- | --- | --- | --- | --- | --- |
| Compound | H37Rv | Mtb-DprE1 ^Y314H^ | Mtb-DprE1 ^Y314C^ | Mtb-DprE1 ^P116S^ | Mtb-MmpL3 ^G253E^ | Δ*tap* | H37Rv | Mtb-DprE1  ^Y314H^ | Mtb-DprE1 ^Y314C^ | Mtb-DprE1 ^P116S^ | Mtb-MmpL3 ^G253E^ | Δ*tap* | CydKO |
| 127-09 | 0.4 | 0.4 | 0.1 | 0.4 | 0.4 | 0.4 | 1.6 | 3.1 | 3.1 | 3.1 | 6.3 | 3.1 | 5 |
| 127-17 | 1.6 | 1.6 | 0.8 | 0.8 | 0.8 | 0.8 | 6.3 | 6.3 | 12.5 | 12.5 | 12.5 | 12.5 | 10 |
| 127-21 | 1.6 | 0.8 | 0.8 | 0.8 | 0.8 | 1.6 | 6.3 | 6.3 | 12.5 | 6.3 | 6.3 | 12.5 | 5 |
| 127-23 | 3.1 | 1.6 | 1.6 | 1.6 | 1.6 | 1.6 | 12.5 | 25 | 12.5 | 25 | 25 | 12.5 | 5 |
| 127-11 | 0.7 | - | - | - | - | 0.8 | 0.1 | - | - | - | - | 0.3 | 0.15 |
| 127-13 | 1.6 | - | - | - | - | 1.6 | 1.6 | - | - | - | - | 3.1 | - |
| 127-14 | 6.3 | - | - | - | - | 12.5 | 6.3 | - | - | - | - | 12.5 | - |
| 127-15 | 0.4 | 0.1 | < 0.05 | 0.1 | 0.2 | 0.8 | 0.1 | 0.2 | 0.2 | 0.2 | 0.4 | 0.4 | 0.62 |
| 127-18 | 25 | - | - | - | - | 12.5 | 25 | - | - | - | - | 12.5 | 5 |
| 127-19 | 0.1 | - | - | - | - | 0.2 | 0.7 | - | - | - | - | 0.8 | 0.62 |
| 127-20 | 1.6^b^ | - | - | - | - | 1.6 | 1.4 | - | - | - | - | 1.6 | 5 |
| 127-22 | 12.5 | - | - | - | - | 12.5 | 1.6 | - | - | - | - | 3.1 | 2.5 |
| MN-6925 | 6.3 | 3.1 | 3.1 | 3.1 | 3.1 | 3.1 | 3.1 | 0.05 | 0.2 | 0.1 | 0.2 | 0.4 | - |
| MN-9483 | 3.1 | - | - | - | - | 3.1 | 1.6 | - | - | - | - | 3.1 | - |

^a^Drug susceptibility testing against DprE1 and MmpL3 mutants was restricted to compounds which exhibited a positive *ini*BAC response

^b^MIC in 7H9 OADC media

- not determined

**REFERENCES**

Abrahams, G.L., Kumar, A., Savvi, S., Hung, A.W., Wen, S., Abell, C., Barry, C.E., 3rd, Sherman, D.R., Boshoff, H.I., and Mizrahi, V. (2012). Pathway-selective sensitization of *Mycobacterium tuberculosis* for target-based whole-cell screening. *Chem Biol* 19**,** 844-854.

Arora, K., Ochoa-Montaño, B., Tsang, P.S., Blundell, T.L., Dawes, S.S., Mizrahi, V., Bayliss, T., Mackenzie, C.J., Cleghorn, L.a.T., Ray, P.C., Wyatt, P.G., Uh, E., Lee, J., Barry, C.E., 3rd, and Boshoff, H.I. (2014). Respiratory flexibility in response to inhibition of cytochrome C oxidase in Mycobacterium tuberculosis. *Antimicrobial agents and chemotherapy* 58**,** 6962-6965.

Chan, K., Knaak, T., Satkamp, L., Humbert, O., Falkow, S., and Ramakrishnan, L. (2002). Complex pattern of Mycobacterium marinum gene expression during long-term granulomatous infection. *Proceedings of the National Academy of Sciences of the United States of America* 99**,** 3920-3925.

Ioerger, T.R., Feng, Y., Ganesula, K., Chen, X., Dobos, K.M., Fortune, S., Jacobs, W.R., Jr., Mizrahi, V., Parish, T., Rubin, E., Sassetti, C., and Sacchettini, J.C. (2010). Variation among genome sequences of H37Rv strains of *Mycobacterium tuberculosis* from multiple laboratories. *J Bacteriol* 192**,** 3645-3653.

Moosa, A., Lamprecht, D.A., Arora, K., Barry, C.E., Boshoff, H.I.M., Ioerger, T.R., Steyn, A.J.C., Mizrahi, V., and Warner, D.F. (2017). Susceptibility of *Mycobacterium tuberculosis* Cytochrome bd Oxidase Mutants to Compounds Targeting the Terminal Respiratory Oxidase, Cytochrome c. *Antimicrobial Agents and Chemotherapy* 61**,** e01338-01317.

Naran, K., Moosa, A., Barry, C.E., 3rd, Boshoff, H.I.M., Mizrahi, V., and Warner, D.F. (2016). Bioluminescent Reporters for Rapid Mechanism of Action Assessment in Tuberculosis Drug Discovery. *Antimicrobial agents and chemotherapy* 60**,** 6748-6757.

Oh, S., Park, Y., Engelhart, C.A., Wallach, J.B., Schnappinger, D., Arora, K., Manikkam, M., Gac, B., Wang, H., Murgolo, N., Olsen, D.B., Goodwin, M., Sutphin, M., Weiner, D.M., Via, L.E., Boshoff, H.I.M., and Barry, C.E., 3rd (2018). Discovery and Structure-Activity-Relationship Study of N-Alkyl-5-hydroxypyrimidinone Carboxamides as Novel Antitubercular Agents Targeting Decaprenylphosphoryl-β-d-ribose 2'-Oxidase. *J Med Chem* 61**,** 9952-9965.

Snapper, S.B., Melton, R.E., Mustafa, S., Kieser, T., and Jacobs, W.R., Jr. (1990). Isolation and characterization of efficient plasmid transformation mutants of Mycobacterium smegmatis. *Mol Microbiol* 4**,** 1911-1919.

Torres, J.N., Paul, L.V., Rodwell, T.C., Victor, T.C., Amallraja, A.M., Elghraoui, A., Goodmanson, A.P., Ramirez-Busby, S.M., Chawla, A., Zadorozhny, V., Streicher, E.M., Sirgel, F.A., Catanzaro, D., Rodrigues, C., Gler, M.T., Crudu, V., Catanzaro, A., and Valafar, F. (2015). Novel katG mutations causing isoniazid resistance in clinical *M. tuberculosis* isolates. *Emerging microbes & infections* 4**,** e42-e42.
